# MPL W515L expression induces TGFβ secretion and leads to an increase in chemokinesis *via* phosphorylation of THOC5

**DOI:** 10.18632/oncotarget.7639

**Published:** 2016-02-23

**Authors:** Anthony D. Whetton, Norhaida Che Azmi, Stella Pearson, Ewa Jaworska, Liqun Zhang, Rognvald Blance, Alexandra C. Kendall, Anna Nicolaou, Samuel Taylor, Andrew J.K. Williamson, Andrew Pierce

**Affiliations:** ^1^ Stem Cell and Leukaemia Proteomics Laboratory, The University of Manchester, Manchester, UK; ^2^ Manchester Pharmacy School, Faculty of Medical and Human Sciences, Manchester Academic Health Science Centre, The University of Manchester, Manchester, UK

**Keywords:** MPLW515L, chemokinesis, THOC5, MYC, S1P

## Abstract

The thrombopoietin receptor (MPL) has been shown to be mutated (MPL W515L) in myelofibrosis and thrombocytosis yet new approaches to treat this disorder are still required. We have previously shown that transcriptome and proteomic effects do not correlate well in oncogene-mediated leukemogenesis. We therefore investigated the effects of MPL W515L using proteomics. The consequences of MPL W515L expression on over 3300 nuclear and 3500 cytoplasmic proteins were assessed using relative quantification mass spectrometry. We demonstrate that MPL W515L expression markedly modulates the CXCL12/CXCR4/CD45 pathway associated with stem and progenitor cell chemotactic movement. We also demonstrated that MPL W515L expressing cells displayed increased chemokinesis which required the MPL W515L-mediated dysregulation of MYC expression via phosphorylation of the RNA transport protein THOC5 on tyrosine 225. In addition MPL W515L expression induced TGFβ secretion which is linked to sphingosine 1-phosphate production and the increased chemokinesis. These studies identify several pathways which offer potential targets for therapeutic intervention in the treatment of MPL W515L-driven malignancy. We validate our approach by showing that CD34+ cells from MPL W515L positive patients display increased chemokinesis and that treatment with a combination of MYC and sphingosine kinase inhibitors leads to the preferential killing of MPL W515L expressing cells.

## INTRODUCTION

Myeloproliferative neoplasms (MPNs) are clonal disorders of hematopoietic stem cells (HSCs) characterized by aberrant proliferation of one or several myeloid lineages. MPNs include essential thrombocythemia (ET), polycythemia vera (PV), and primary myelofibrosis (PMF). Whilst these disorders have overlapping clinical features it is only in recent years that the molecular basis of these diseases have been defined [1]. The W515L mutation in the thrombopoietin receptor, MPL, occurs in around 10% of ET and PMF patients. [2] Furthermore, as PV progresses bone marrow scarring can occur leading to myelofibrosis (MF) in 5-15% of cases [3]. MF arises *via* a poorly understood process but results in bone marrow failure [4]. Whilst the median survival for patients with PV is more than 10 years [5] that for MF is only five years. [6] As well as the onset of MF patients with MPN can progress to acute myeloid leukemia (AML). [7] Thus a consideration of the effects of MPL W515L will inform our understanding of MF and leukemic progression. This could lead to effective management of the disease.

In MPNs HSCs are thought to secrete factors that activate fibroblasts in the bone marrow, TGFβ being one such factor [8] and this has been reported to promote MF and myeloproliferation, both hallmarks of MF. [9] TGFβ induced liver fibrosis has been shown to be related to intracellular sphingosine 1-phosphate (S1P) levels. [10] S1P can bind to a cognate receptor to elicit signal transduction in HSCs [11] which has differential effects on the motility of HSC and more mature populations in the bone marrow. [12]

We have published that there is a poor correlation between oncogene-mediated mRNA and proteome changes. [13, 14] Therefore we analysed the effects of the MPL W515L using proteomics. The aim was to identify the downstream effectors of MPL W515L that may offer opportunities for therapeutic intervention. We demonstrate that MPL W515L expression leads to an increase in proteins associated with motility and that chemokinesis is increased in these cells. MPL W515L-induced phosphorylation of the spliceosome protein THOC5 is critical in this process. We also show that the THOC5 induced effects on chemokinesis are reliant on MYC signalling and S1P effectors. The observations on motility were validated in primary patient material and we demonstrate the potential therapeutic value of disruption of MYC and S1P.

## RESULTS

### Analysis of MPL W515L effects

To gain an understanding of the mechanisms of MPL W515L induced effects we undertook a proteomic investigation. The MPL W515L transfected cell line was shown to be independent of Interleukin-3 (normally required for survival and proliferation of Ba/F3 cells) and to have the same growth rate as control cells cultured in Interleukin-3 (Supplementary Figure 2A-2B). The workflow for the mass spectrometric analysis is illustrated in Supplementary Figure 2C. Replicate samples were present in each of the three experiments to allow the calculation of the values defining a change in protein level ensuring only high confidence changes were considered. [13, 15] We defined a protein level as changing where a protein has an isobaric tag reporter ion-based quantification ratio outside the range in which 95% of protein ratios for the internal replicate are found and a p-value of 0.05 or less. This “significance interval” was determined for each experimental run and accounts for the technical and biological variation seen in each run (see Supplementary Table 2).

Cellular fractionation was undertaken (Supplementary Figure 2D) to allow improved data acquisition and quantification of cytosolic and nuclear proteins. [15, 16] As previously reported the expression of leukemogenic oncogenes did not affect the cellular protein content [13] and the average nuclear to cytoplasmic protein content ratio was 1:3.5 +/−0.2 (mean+/−SEM). Thus 100μg of each cell population was used for isobaric tag labelling with no normalisation required for protein content differences. We identified 3392 nuclear proteins (Supplementary Table 3) and 3550 cytoplasmic proteins (Supplementary Table 4) with associated isobaric tag quantification (3469 and 3922 proteins in total). The false discovery rate was 0.14% for the nuclear fraction and 0.08% for the cytoplasmic fraction.

### The effect of MPL W515L on the nuclear proteome

Of the nuclear proteins quantified 27 were shown to change as a consequence of MPL W515L expression (Table 1). Within the proteins shown to change there was evidence for perturbation of the RAS pathway in that both JUN b and Traf3ip3 change in expression. In a previous study looking for commonalities in the action of six different leukemogenic tyrosine kinases we showed that disruption of DNA mismatch repair to be a common feature [15]. The data presented here also shows potential disruption of DNA repair in that both MDC1 and MSH6 expression levels are altered by MPL W515L. The changes in Cnot7 have links to the post translational regulation of THOC5 a spliceosome protein known to be a common downstream phosphorylation target of numerous leukemogenic oncogenes. [17]

**Table 1:** Nuclear proteins whose expression is altered by the expression of MPLW515L

Accession	Gene Symbol	Protein Name	Significance	Ratio
ENSMUSP00000080949	Mdc1	Mediator of DNA damage checkpoint protein 1	0.99	1.62
ENSMUSP00000087947	Fyb	FYN-binding protein (FYN-T-binding protein	0.99	1.65
ENSMUSP00000023074	Parvg	Gamma-parvin	−0.99	0.54
ENSMUSP00000109325	Tpm1	Tpm1 protein	0.99	1.62
ENSMUSP00000029941	Pdlim5	PDZ and LIM domain protein 5	−0.95	0.69
ENSMUSP00000028059	Rsu1	Ras suppressor protein 1 (Rsu-1)(RSP-1)	0.98	1.43
ENSMUSP00000099375	Itga2b	Integrin alpha-IIb Precursor	0.99	1.78
ENSMUSP00000005503	Msh6	DNA mismatch repair protein Msh6	−0.96	0.76
ENSMUSP00000015581	Gzmb	Granzyme B(G,H) Precursor	−0.99	0.53
ENSMUSP00000077342	Skap2	Src kinase-associated phosphoprotein 2	0.98	1.68
ENSMUSP00000118997	Cbfa2t3	Core-binding factor, runt domain, alpha subunit 2	0.98	1.47
ENSMUSP00000040977	Traf3ip3	TRAF3-interacting JNK-activating modulator	−0.97	0.7
ENSMUSP00000095286	Dock10	Dedicator of cytokinesis protein 10 (Zizimin-3)	−0.99	0.53
ENSMUSP00000101921	Sept1	Putative uncharacterized protein Sept1	−0.98	0.63
ENSMUSP00000117606	Fcho1	Putative uncharacterized protein Fcho1	−0.96	0.7
ENSMUSP00000064680	Junb	Transcription factor jun-B (MyD21)	0.96	1.48
ENSMUSP00000106311	Pitrm1	Presequence protease, mitochondrial Precursor	0.98	1.77
ENSMUSP00000110707	Fam107b	Protein FAM107B	−0.99	0.48
ENSMUSP00000073124	Plec	Plectin-1 (Plectin-6)(PLTN)(PCN)	−0.99	0.68
ENSMUSP00000117304	Cnot7	CCR4-NOT transcription complex, subunit 7, isoform	0.97	1.68
ENSMUSP00000039776	Pold1	DNA polymerase delta catalytic subunit	−0.92	0.8
ENSMUSP00000030684	Gnl2	Nucleolar GTP-binding protein 2	0.93	1.28
ENSMUSP00000030030	Tex10	Testis-expressed sequence 10 protein	0.94	1.28
ENSMUSP00000067685	Iqgap2	Ras GTPase-activating-like protein IQGAP2	−0.93	0.73
ENSMUSP00000074216	Gm8894	Myosin light polypeptide 6	−0.95	0.62
ENSMUSP00000103267	Ldha	L-lactate dehydrogenase A chain (LDH-A)	−0.91	0.7
ENSMUSP00000082682	Hmgb1	High mobility group protein B1	−0.9	0.71

Proteins shown are those that are defined as changing by the parameters outlined in Results section 1. “ratio” represents an average of the MPL W515L:MSCV protein ratios across multiple experiments. This is supported by the averaged confidence across experiments, “significance”, which indicates how closely the experiments agree. For inclusion the “significance” level was 0.90 or above.

**Figure 1:**
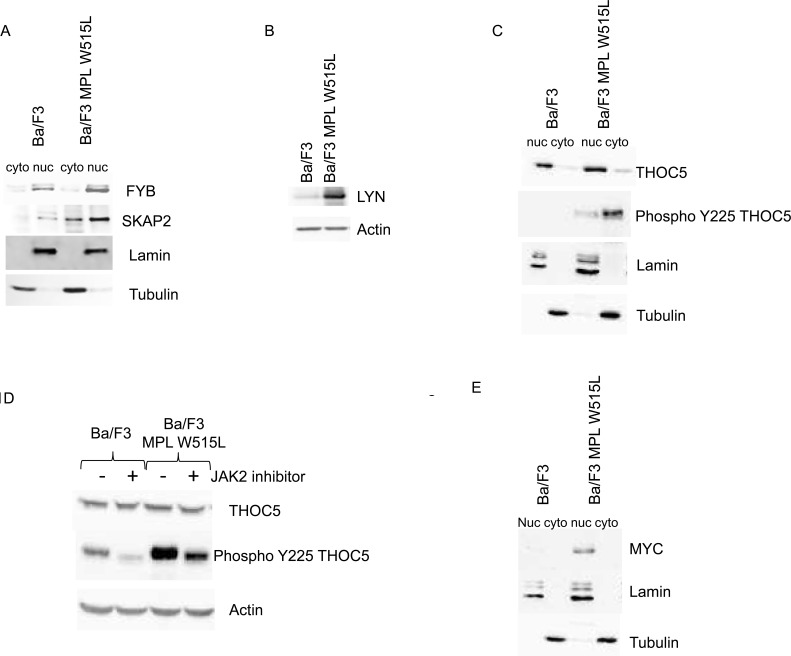

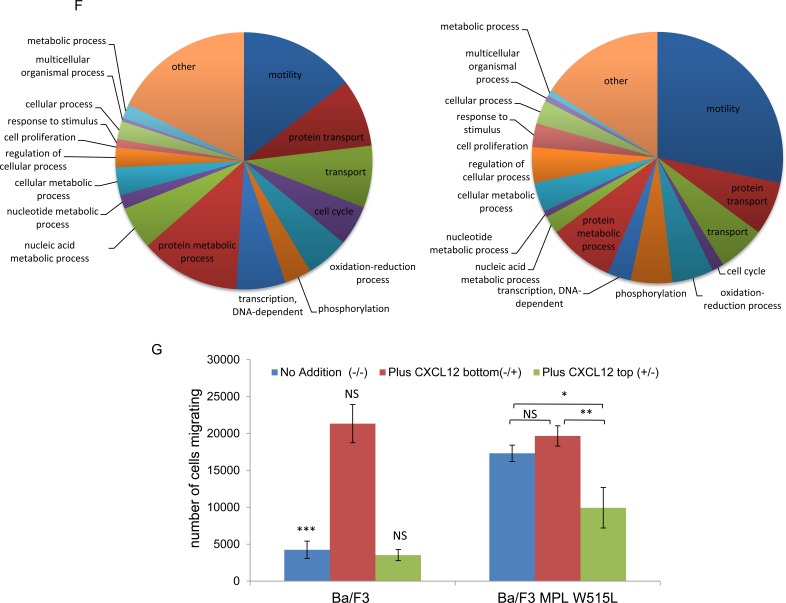
The effect of MPL W515L on the Nuclear and cytoplasmic proteome Control and MPL W515L expressing cells were subject to nuclear/cytoplasmic fractionation and the levels and distribution of the proteins indicated assessed by western blot analysis. (**A**, **C** and **E**). Lamin and tubulin expression were used as loading controls and fractionation markers. **B.** The expression level of Lyn was assessed by western blot in whole cell lysates. Actin was used as a loading control. **D.** Western blot analysis of THOC5 phosphorylation in control and MPL W515L expressing cells following treatment with 0.5μM of the Jak2 inhibitor Ruxolitinib for 16hours. Actin was used as a loading control. **F.** Pie charts of cytoplasmic proteins categorised by biological-process are shown. The left panel represents all the proteins identified (3447) with an assigned biological process and the right panel those defined as changing (131) in at least two of the three replicates. **G.** The CXCL12 induced chemotactic response of control and MPL W515L expressing cells was assessed in Boyden chamber assays. Cells (1×10^5^) were added to the top well. CXCL12 (200ng/ml) was added to bottom well (−/+), top well (+/−) or omitted (−/−). The number of cells in the bottom well was counted after 6 hours. Results are the mean ± SEM, *n* = 4. The results of a t-test between Ba/F3 and MPL W515L are shown above the results for Ba/F3 and between the treatments for the MPL W515L. The results of the t-test are represented by; NS non-significant, * < 0.05, ** < 0.01, *** < 0.001.

The proteomic analysis also showed that FYB was up-regulated in MPL W515L expressing cells. FYB is known to bind SRC family members and prevent the degradation of SKAP2. The data also indicate an increase in SKAP2 expression. The up regulation of FYB and SKAP2 were confirmed by western blot analysis (Figure 1A) verifying the robust nature of iTRAQ proteome data as we have previously shown. [13, 15, 18] The disruption of the SRC signalling pathway, as indicated by the up-regulation of FYB and SKAP2, was investigated further by qRT-PCR of SRC family members. The results of the PCR (data not shown) suggested an up regulation of the SRC kinase family member LYN in MPL W515L expressing cells. This up regulation of LYN expression was confirmed by western blot (Figure 1B). We have previously shown that SRC family proteins stimulate the phosphorylation of THOC5 on tyrosine Y225 [19]. This along with the changes in Cnot7 (see above) led us to investigate the status of THOC expression and phosphorylation in MPL W515L expressing cells. Whilst the expression of THOC was unaffected by MPL W515L expression the degree of Y225 phosphorylation was markedly increased (Figure 1C). THOC5 shuttles between the nucleus and cytosol but the Y225 phosphorylated THOC5 has a mainly cytosolic location (Figure 1C). This MPL W515L enhanced THOC5 phosphorylation was modulated by the JAK2 inhibitor ruxolotinib which reduced the level of THOC5 Y225 phosphorylation (Figure 1D). Analysis of transcription factors with binding motifs present in the promoter region of genes for those proteins that changed in expression in response to MPL W515L revealed that MYC consensus binding sites were associated with a number of genes whose proteins increased in expression. This tentative observation and the fact that THOC5 has been shown to regulate MYC expression [20] led us to investigated whether MPL W515L had any effect on MYC expression levels. Western blot analysis of MYC levels showed an increase when MPL W515L was present and that MYC had a mainly nuclear distribution (Figure 1E).

### The effect of MPL W515L on the cytoplasmic proteome

From the comprehensive list of proteins quantified in the cytoplasmic fractions 42 were designated as changing as a consequence of MPL W515L expression (Table 2). In addition to the JAK/STAT pathway MPL activation has been shown to involve the RAS/RAF/MAPK pathway and it has been demonstrated that RAS and RAP1 are needed for sustained ERK activation in MPL signalling. [21] In line with these observations, and agreement with perturbation of the RAS pathway in our nuclear proteome analysis, four of the proteins identified as changing as a consequence of MPL W515L expression in the cytoplasm are constituents of the RAS pathway.

**Table 2:** Cytoplasmic proteins whose expression is altered by the expression of MPLW515L

Accession	Gene Symbol	Protein Name	Significance	Ratio
ENSMUSP00000028059	Rsu1	Ras suppressor protein 1	0.98	1.93
ENSMUSP00000099375	Itga2b	Integrin alpha-IIb Precursor	0.99	2.44
ENSMUSP00000021611	Pitrm1	Presequence protease, mitochondrial Precursor	0.98	1.82
ENSMUSP00000021028	Itgb3	Integrin beta-3 Precursor	0.99	2.60
ENSMUSP00000003017	Tbxas1	Thromboxane-A synthase	0.98	2.23
ENSMUSP00000121201	Lcp1	Putative uncharacterized protein Lcp1	−0.97	0.57
ENSMUSP00000002640	Scin	Adseverin (Scinderin)	−0.99	0.41
ENSMUSP00000003912	Calr	Calreticulin Precursor	−0.96	0.64
ENSMUSP00000110707	Fam107b	Protein FAM107B	−0.98	0.46
ENSMUSP00000025762	Banf1	Barrier-to-autointegration factor	−0.97	0.56
ENSMUSP00000086795	Lgals1	Galectin-1 (Gal-1)(Lectin galactoside-binding soluble 1)	−0.99	0.28
ENSMUSP00000075690	Serpinb1a	Leukocyte elastase inhibitor A	−0.98	0.54
ENSMUSP00000028239	Gsn	Gelsolin Precursor	−0.97	0.54
ENSMUSP00000027645	Ptprc	Receptor-type tyrosine-protein phosphatase C Precursor	−0.98	0.52
ENSMUSP00000084882	Gda	Guanine deaminase (Guanase)(Guanine aminase)	−0.99	0.42
ENSMUSP00000043724	Rcsd1	CapZ-interacting protein (Protein kinase substrate CapZIP)	−0.98	0.52
ENSMUSP00000025207	Tmem173	Transmembrane protein 173	−0.99	0.41
ENSMUSP00000066238	Rap1b	Ras-related protein Rap-1b Precursor	0.97	1.86
ENSMUSP00000070427	Zyx	Zyxin	0.97	1.79
ENSMUSP00000070113	Nrgn	Neurogranin	0.95	1.63
ENSMUSP00000097772	AC123724.1	Putative uncharacterized protein Bin2	0.96	1.60
ENSMUSP00000104825	Ifi47	Interferon gamma inducible protein 47	0.96	1.69
ENSMUSP00000020529	Ahsa2	Activator of 90 kDa heat shock protein ATPase homolog 2	0.96	1.68
ENSMUSP00000052020	Flnb	Filamin-B	−0.96	0.63
ENSMUSP00000114705	Dbi	Diazepam binding inhibitor	−0.95	0.65
ENSMUSP00000025904	Prdx5	Peroxiredoxin-5, mitochondrial Precursor	−0.95	0.64
ENSMUSP00000097154	Tjp2	Tight junction protein ZO-2	−0.96	0.62
ENSMUSP00000063825	Pcx	Pyruvate carboxylase, mitochondrial Precursor	−0.96	0.63
ENSMUSP00000085253	Rab44	Ras-related protein Rab-44	−0.98	0.50
ENSMUSP00000099853	Cd47	Leukocyte surface antigen CD47 Precursor	−0.98	0.49
ENSMUSP00000073975	Pdcd4	Programmed cell death protein 4	−0.98	0.40
ENSMUSP00000101657	Ifitm1	Interferon induced transmembrane protein 1	−0.99	0.33
ENSMUSP00000039797	Prkar2b	cAMP-dependent protein kinase type II-beta regulatory subunit	0.94	1.44
ENSMUSP00000105647	Lgmn	Legumain Precursor	0.92	1.50
ENSMUSP00000023486	Tfrc	Transferrin receptor protein 1	0.91	1.47
ENSMUSP00000032949	Coro1a	Coronin-1A	−0.95	0.67
ENSMUSP00000109707	Capg	Macrophage-capping protein	−0.95	0.67
ENSMUSP00000067685	Iqgap2	Ras GTPase-activating-like protein IQGAP2	−0.91	0.69
ENSMUSP00000072840	Sh3kbp1	SH3 domain-containing kinase-binding protein 1	−0.92	0.67
ENSMUSP00000103007	Idh2	Isocitrate dehydrogenase [NADP]	−0.95	0.66
ENSMUSP00000040956	Sccpdh	Probable saccharopine dehydrogenase	−0.94	0.68
ENSMUSP00000116616	Tbcel	Putative uncharacterized protein Tbcel	−0.92	0.66

Proteins shown are those that are defined as changing as in Table 1.

When all the proteins identified and those designated as changing were assigned to a biological process a specific enrichment of proteins involved in motility (15% to 28%) was seen in the proteins shown to be changing (Figure 1F). This is of interest since MPNs are often characterised by a large increase in the number of circulating CD34^+^ cells indicating that adhesion within or egress from the stem cell niche is disrupted. We therefore asked the question whether MPL W515L expression led to a modulation of cellular motility. Whilst the MPL W515L expressing cells displayed no change in their chemotactic response to CXCL12 (*p* = 0.13) they showed a 4.1 fold increase in chemokinesis in the absence of any external stimulus (*p* < 0.001) (Figure 1G). Thus the changes in expression of the motility proteins we observed are associated with a change in a biological parameter relevant in blood cell production/function and leukemogenesis.

### The mechanism for the effect of MPL W515L on motile response

**Figure 2:**
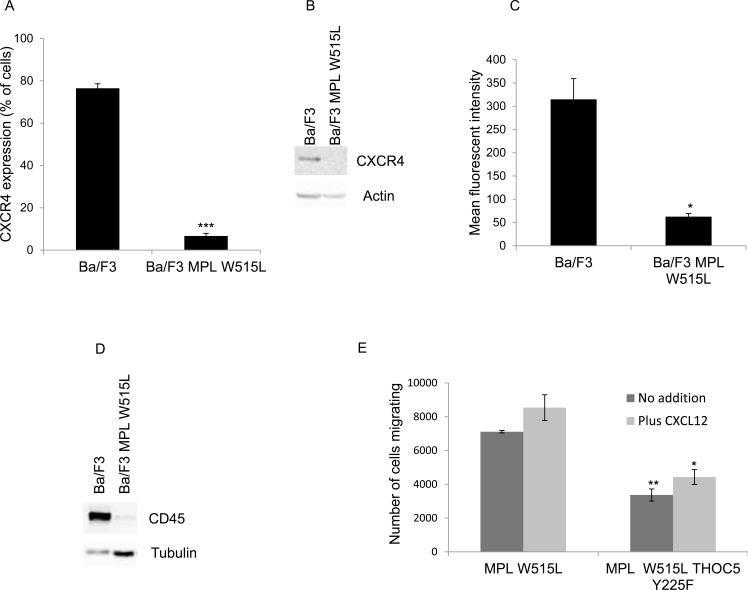
THOC5 plays a role in MPL W515L induced motility **A.** Cell surface expression of CXCR4 was assessed using flow cytometry. Results are expressed as the number of positively staining cells +/−SEM (*n* = 3). **B.** Western blot analysis of CXCR4 expression in whole cell lysates. Actin was used as a loading control. **C.** Cell surface expression of CD45 was assessed using flow cytometry. Results are expressed as the mean fluorescence intensity +/−SEM (*n* = 3). **D.** Western blot analysis of CD45 expression in whole cell lysates. Tubulin was used as a loading control. **E.** Chemokinesis was measured using Boyden chamber assays in parental Ba/F3 cells, MPL W515L expressing cells and MPL W515L co-transfected with THOC5 Y225F. The results of a t-test between Ba/F3 and MPL W515L (A, B) or MPL W515L and MPL W515L THOC5 Y225F (E) are shown and represented by; * < 0.05, ** < 0.01, *** < 0.001.

The perturbation in chemotactic response is in keeping with the pathology of MPNs [22]. We therefore investigated this phenomenon in greater detail to understand the mechanism by which this was achieved. CD45 has been reported to be a JAK2 phosphatase [23] and our data indicated a downregulation of CD45 in MPL W515L expressing cells (Table 2). We therefore investigated the CXCL12/CXCR4/CD45 pathway in an attempt to understand the potential mechanism of motility stimulus. Surprisingly, given the increase in chemokinesis, both CXCR4 and CD45 levels (in agreement with our proteomic data) were reduced in the MPL W515L expressing cells (Figure 2A-2D). The number of cells expressing cell surface CXCR4 was vastly reduced (Figure 2A) and this was reflected in a reduced overall level of expression as assessed by western blot on whole cell lysates (Figure 2B). Whilst all cells expressed CD45 the amount of cell surface CD45 was reduced (Figure 2C) and again this was reflected in a reduced overall level of expression (Figure 2D and Table 2).

THOC5 a target for MPL W515L (Figure 1C, 1D) is an mRNA processing protein that lies downstream of the CXCL12/CD45 signalling pathway and its phosphorylation can affect motility. [19] Therefore we next determined whether THOC5 phosphorylation and increased MYC expression played any role in the enhanced chemokinetic behaviour of the MPL W515L expressing cells. We have previously shown that knockdown of THOC5 expression causes apoptosis [24] which negates the use of such techniques in the study of the effects of THOC5 phosphorylation. We therefore chose to express a phospho-mutant of THOC, Y225F, in the MPL W515L cells to investigate whether this had any dominant negative effects on the increased chemokinesis. THOC5 Y225F displayed a similar nuclear/cytoplasmic localisation/ratio to endogenous THOC5 (Supplementary Figure 3A) and did not appear to influence endogenous THOC5 expression or phosphorylation. However THOC5 Y225F expression in the MPL W515L cells led to a loss of both the enhanced oncogene mediated chemokinesis and the cellular ability to respond to CXCL12 (Figure 2E).

**Figure 3:**
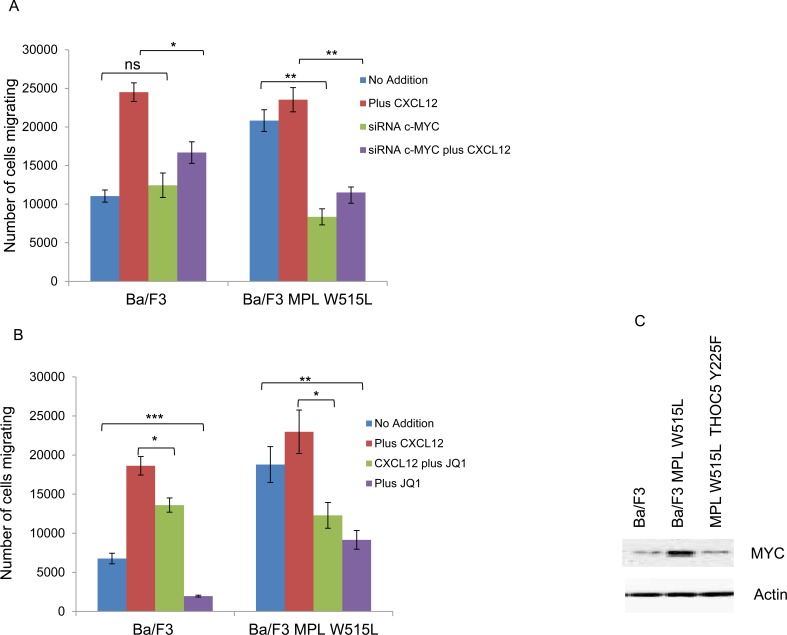

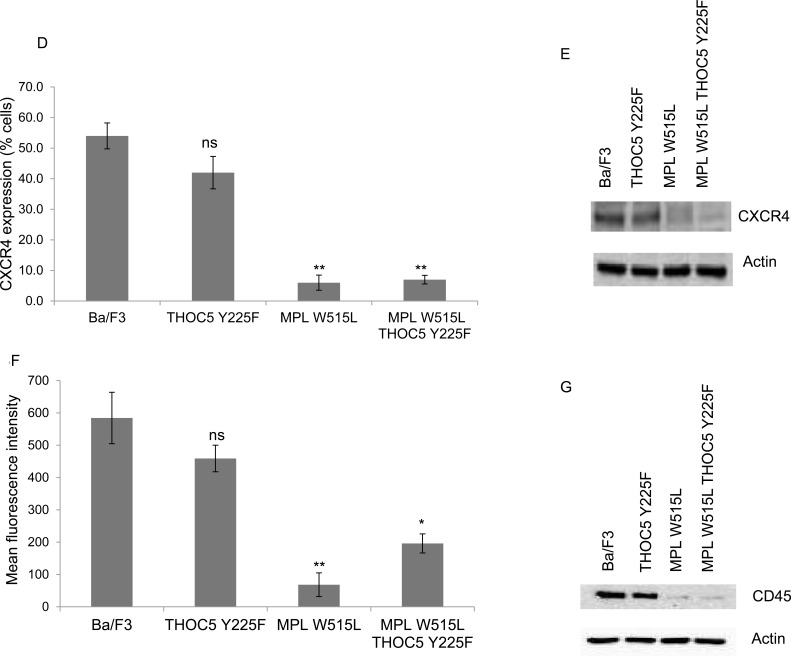
MYC plays a role in MPL W515L induced motility Chemokinesis and chemotaxis was measured using Boyden chamber assays in parental Ba/F3 cells and MPL W515L expressing cells 24 hours post transfection with c-MYC SiRNA **A.** or following 2 hours pre-incubation with 500nM of the MYC inhibitor JQI **B.** Results shown are the number of cells migrating (mean ± SEM, *n* = 3), cell viability was greater than 94% post migration assay. **C.** Western blot analysis of MYC expression with actin as a loading control. **D.** Cell surface expression of CXCR4 was assessed using flow cytometry. Results are expressed as the number of positively staining cells +/−SEM (*n* = 4). **E.** Western blot analysis of CXCR4 expression in whole cell lysates. Actin was used as a loading control. **F.** Cell surface expression of CD45 was assessed using flow cytometry. Results are expressed as the mean fluorescence intensity +/−SEM (*n* = 3). **G.** Western blot analysis of CD45 expression in whole cell lysates. Tubulin was used as a loading control. The results of a t-test against Ba/F3 (D, F) or as shown (A, B) are shown and represented by; * < 0.05, ** < 0.01, *** < 0.001.

The next question we investigated was whether the increase in chemokinesis was *via* the modulation of MYC. Either knockdown of MYC using siRNA (Figure 3A) or the inhibition of MYC with the BET bromodomain inhibitor JQ1 (Figure 3B) led to a reduction in the chemokinetic enhancement induced by MPL W515L. Both siRNA and JQ1 treatment led to a significant down regulation of MYC (Supplementary Figure 3B, 3C). Given our previous observations on the role of THOC5 in MYC regulation [20] and the apparent role of THOC5 and MYC in the MPL W515L-induced chemokinesis we hypothesised that the phosphorylation of THOC5 at Y225 achieved its effects on motility *via* the modulation of MYC expression. Western blot analysis of MYC expression in the MPL W515L cells transfected with THOC5 Y225F clearly shows a down regulation of MYC protein levels (Figure 3C). This suggested a signalling cascade existed from MPL W515L which led to THOC5 phosphorylation altering MYC expression contributing to increased motility. The next question we addressed was whether THOC5 Y225 phosphorylation has any effect on the MPL W515L induced decrease in CXCR4 and CD45 expression. Flow cytometric analysis and western blot assessment of CXCR4 and CD45 expression in THOC5 Y225F transfected cells suggested that whilst THOC5 phosphorylation may be downstream of these proteins a feedback loop does not exist as it does not lead to any alteration in CXCR4 or CD45 expression (Figure 3D-3G).

### TGFβ and S1P are involved in the MPL W515L induced chemotaxis

**Figure 4:**
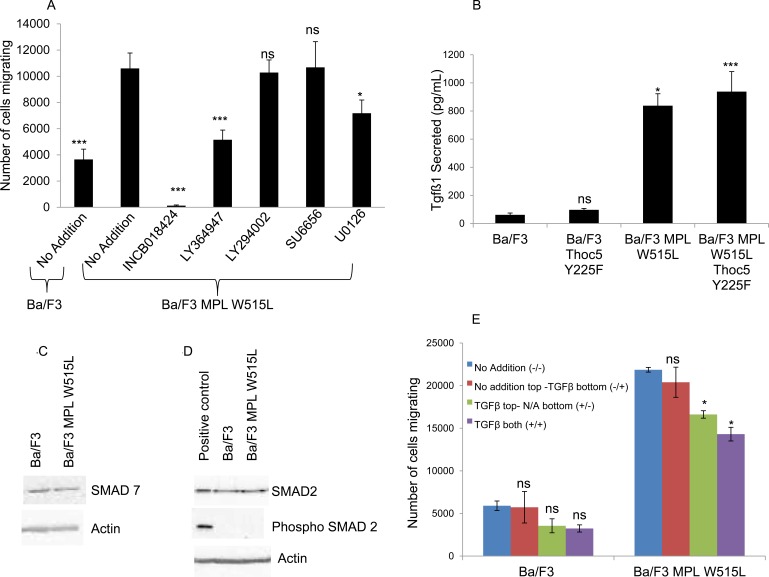
A role for TGFβ-1 in the MPL W515L induced chemotaxis **A.** The effect of inhibitor treatment on chemokinesis in MPL W515L was assessed. Cells were pre-incubated with inhibitors for 2 hours before undertaking the motility assay. 10^5^ cells were used and the number of cells migrating into the bottom well counted after 6 hours incubation. Results are the mean ± SEM of at least three experiments. Inhibitors used were 50 μM INCB018424 (JAK2 inhibitor), 5μM LY364947 (TGFβ inhibitor), 10μM LY294002 (PI3K inhibitor), 20μM SU6656 (Src inhibitor) and 10μM U0126 (MEK 1/2 inhibitor). Cell viability was greater than 90% prior to and post the migration assay. **B.** The levels of TGFβ-1 were measured in culture supernatants from parental Ba/F3, MPL W515L, THOC5 Y225F and MPL W515L co-transfected with THOC5 Y225F expressing cells using the Quantikine ELISA from R&D systems. Results are displayed as pg/ml of cell culture supernatant +/−SEM (*n* = 3). The levels of SMAD7 **C.**, SMAD2 and phospho S465/467 SMAD2 **D.** expression in Ba/F3 and MPL W515L expressing cells was assessed by western blot. **E.** Chemokinesis was measured using Boyden chamber assays in parental Ba/F3 cells and MPL W515L expressing cells with 5ng/ml TGFβ added to either the top well (+/−) bottom well (−/+) or both wells (+/+). Results shown are the number of cells migrating (mean ± SEM, *n* = 3). The results of a t-test against MPL W515L no addition (A) Ba/F3 (B) or no addition (E) are shown and represented by; * < 0.05, ** < 0.01, *** < 0.001.

To further delineate the pathways involved in this MPL W515L - THOC5 - MYC enhancement of motility we undertook a screen of signal transduction inhibitors to gain insight into which pathways may be up-regulated so contributing to the increased motility (Figure 4A). It has been reported that MPL W515L leads to the constitutive activation of the PI3K and ERK pathway [25, 26] and that Src is involved in motility signalling *via* the CXCR4/CD45 axis. [27, 28] We therefore chose inhibitors to these pathways. JAK2 inhibitor ruxolitinib (INCB018424) can be seen to totally abolish any chemokinectic behaviour. Of the inhibitors employed only MEK (U0126) and TGFβ (LY364947) inhibition had any significant effect on MPL W515L-mediated chemokinesis.

Interestingly we have previously reported that TGFβ protein level is up-regulated by leukemogenic oncogenes. [15] There was also an indication in one LCMSMS iTRAQ experiment that this cytokine was elevated by MPL W515L expression. Given this data and TGFβ's importance in CML pathogenesis, [8] MPN fibrosis [9, 29] and motility [30] we investigated the levels of TGFβ in the extracellular medium. ELISA based immunoassays of secreted TGFβ showed a marked increase in conditioned media from MPL W515L expressing cells (Figure 4B). Perhaps somewhat surprisingly the secretion of TGFβ does not appear to act in a paracrine fashion failing to activate the classical TGFβ signalling pathways with no change in expression of either SMAD 7 (Figure 4C) or phosphorylation of SMAD 2 (Figure 4D).

**Figure 5:**
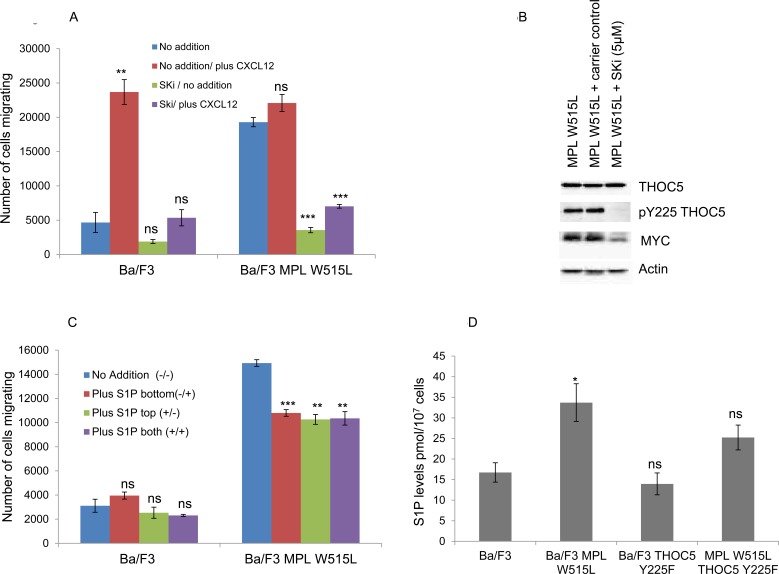
The role of S1P in the MPL W515L-induced chemotaxis **A.** Chemokinesis was measured using Boyden chamber assays in parental Ba/F3 cells and MPL W515L expressing cells following 2 hours pre-incubation with 10μM sphingosine kinase inhibitor SKi. Results shown are the number of cells migrating (mean ± SEM, *n* = 3). **B.** Assessment of THOC5 phosphorylation and MYC expression by western blot analysis in Ba/F3 and Ba/F3 cells expressing MPL W515L following 6 hours treatment with 10μM sphingosine kinase inhibitor SKi. **C.** Chemokinesis was measured using Boyden chamber assays in parental Ba/F3 cells and MPL W515L expressing cells with 5μg/ml S1P added to either the top well (+/−) bottom well (−/+) or both wells (+/+). Results shown are the number of cells migrating (mean ± SEM, *n* = 3). **D.** Levels of intracellular S1P in the cell lines indicated were measured by Mass Spectrometry. Results shown are expressed as pmol per 10^7^ cells (mean ± SEM, *n* = 6). The results of a t-test between treated and untreated are shown and represented by; * < 0.05, ** < 0.01, *** < 0.001.

Does this increase in TGFβ play a role in the enhanced chemokinesis? Despite showing a decrease in the enhanced chemokinesis the MPL W515L Ba/F3 cells transfected with the phospho mutant of THOC5, THOC5 Y225F, still produce TGFβ to a comparable level to that of the MPL W515L Ba/F3 cell line (Figure 4B). Also TGFβ does not display any chemotactic properties on the Ba/F3 cells (Figure 4E). These results offer a paradox given that we have already shown that inhibition of TGFβ led to a partial reduction in the chemokinesis in the MPL W515L expressing cells (Figure 4A). A possible explanation would be that the TGFβ may activate a secondary pathway in the MPL W515L expressing cells. TGFβ signalling has been shown to interact with that of S1P [31] and also to activate S1P release. [32, 33] In addition sphingosine kinase has been reported to play a role in mediating TGFβ enhanced migration of both breast and esophageal cancer cells. [34, 35] Given the importance of S1P in the egress of HSCs from the bone marrow [11, 36] and our previous data on the differential effects of S1P on the motility of primitive haemopoietic cells [12] we investigated the possibility that the TGFβ effects were mediated *via* S1P. Interference of S1P production *via* inhibition of sphingosine kinase completely blocked the enhanced chemokinesis seen in the MPL W515L expressing cells (Figure 5A) and in accordance with our observations on the role of THOC5 and MYC led to a dramatic decrease in the level of THOC5 Y225 phosphorylation and MYC expression (Figure 5B). This is despite the fact that S1P added exogenously did not act as a chemo-attractant for either Ba/F3 or MPL W515L expressing Ba/F3 cells (Figure 5C) or lead to the phosphorylation of THOC5 (Supplementary Figure 4). Yu et al have previously reported that whilst extracellular S1P is chemo-attractant intracellular S1P acts to induce non-directional cell movement. [37] In accordance with the report of Yu et al measurement of intracellular S1P levels indicate that the MPL W515L expressing cells have increased levels of this phospholipid (Figure 5D). Transfection of the MPL W515L cells with the THOC mutant Y225F reduced the level of S1P (Figure 5D) which is in line with the reduction in chemokinesis observed in these cells (Figure 2E).

The Ba/F3 cell study has provided insights into the downstream effectors of MPL W515L. This analysis was not possible using primary cell material. We then considered the relevance of these data to primary MPL W515L positive cells.

### Confirmation of cell line observations in patient material

**Figure 6:**
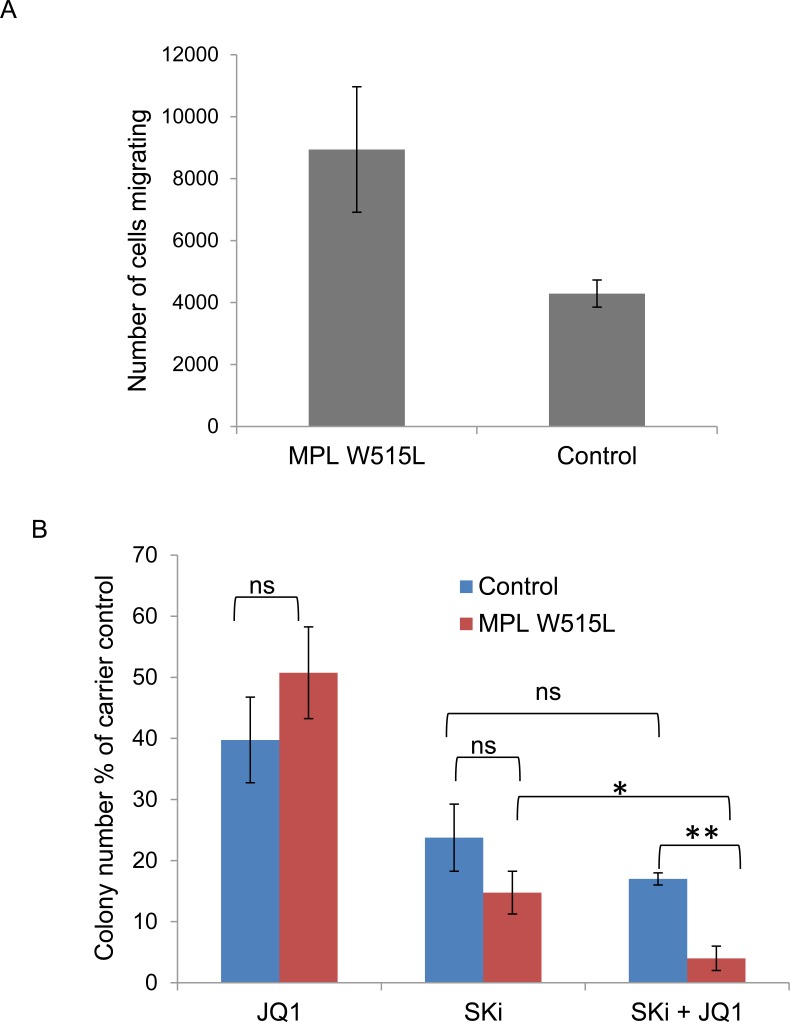
Confirmation of cell line observations in primary cells **A.** Chemokinesis of CD34+ cells from MPL W515L positive Essential Thrombocythemia patients and non-leukemic patients was assessed using Boyden chamber assays in 96-transwell plates. 3 × 10^4^ CD34+ cells in 30μl of media (Fischers 10% v/v HS) were placed in the top well and 30μl of media in the bottom wells. Plates were incubated at 37°C for 6 hours and the number of cells migrating into the bottom well were counted. Results shown are the number of cells migrating (mean+/−SEM, *n* = 3). **B.** The effect of MYC inhibition (250nM JQ1) and sphingosine kinase inhibition (10 μM SKi) on the ability of CD34+ cells from MPL W515L positive Essential Thrombocythemia patients and non-leukaemic patients to form colonies in methylcellulose was assessed. Data is displayed as the total number of colonies expressed as a percentage of the carrier control (mean+/−SEM, *n* = 4).

Despite the extremely limited availability of clinical material from patients with the MPL W515L mutation we succeeded in obtaining samples to verify our cell line based observations in primary patient material. Given the scarcity of samples we chose to verify the downstream effects of the protein changes rather than the protein changes themselves. We first asked the question whether MPL W515L positive patient cells displayed increased chemokinetic behaviour. This was indeed the case with CD34+ cells of MPL W515L positive patients demonstrating increased chemokinesis compared to non-leukemic CD34+ cells (Figure 6A). We next undertook an investigation into whether any of the perturbed pathways we had identified offered any potential as therapeutic targets. As the ultimate goal of any therapy is elimination of the leukemic repopulating cell we assessed the effects of inhibition of both MYC and sphingosine kinase on CD34+ cells in colony forming assays. The inhibitors chosen, JQ1 and Ski, or derivatives of these inhibitors are already in clinical trial for the treatment of other cancers which would aid repositioning of the drugs for use in the treatment of MPL W515L driven leukaemia. Given the extremely limited availability of patient material we tested the drugs for their dose related effects using cell lines then used the optimal dose for the work described below. Whilst individually the drugs did not have a significant difference in terms of inhibition of colony formation the combination appears to promote preferential killing of the leukemic cells (Figure 6B).

## DISCUSSION

Leukemias and myeloproliferative disorders are associated with mutated genes in signal transduction pathways. These offer targets to treat leukemias and successful approaches have been developed which have in the form of tyrosine kinase inhibitor based therapies for diseases such as CML. [38] The identification of activating mutations in the thrombopoietin receptor gene (MPL W515L) in myelofibrosis thus afforded opportunities to define downstream targets for therapeutic intervention. A cautionary point, however is that although MPL W515L activates JAK2 the use of inhibitors to JAK2 does not reduce mutant allele burden [39, 40] and as such have been argued to offer little improvement on chemotherapeutic agents (such as hydroxyurea) in MPNs [41]. Hence, understanding the effectors of MPL more fully is important for the development of a firm knowledge base on the molecular pathogenesis of MF thereby leading to opportunities for increased translational research. In this respect we have produced a comprehensive data set on the proteomics effects of MPL W515L.

**Figure 7:**
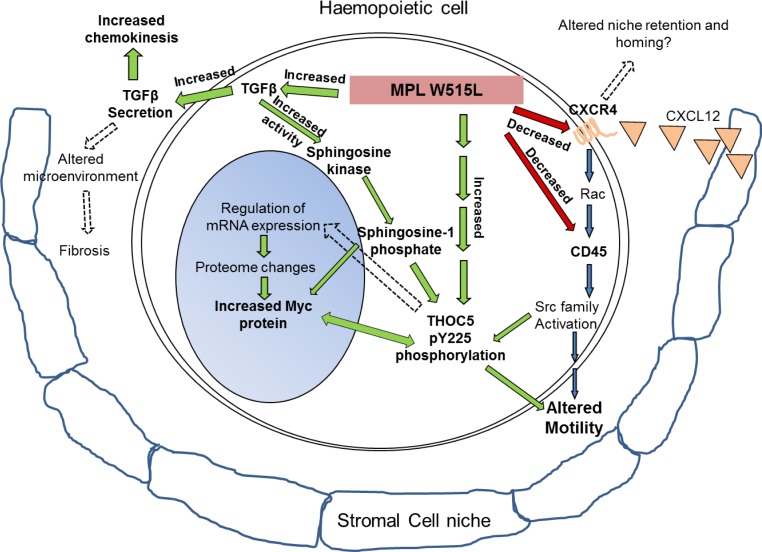
MPL W515L induced pathways: Schematic representation of the results depicting the MPL W515L induced protein and phenotypic changes Red/green arrows and bold text represent observations from this study and blue arrows and normal text recognised pathways. In addition broken arrows depict hypothesised effects.

We observed that MPL W515L potentiated expression of proteins involved in motility and disrupted the CXCL12/CXCR4/CD45/SRC/THOC5 pathway (Summarised in Figure 7). We have clearly shown chemokinesis is increased not only in our model but also in primary cells from patients with the MPL W515L mutation. This observation fits with the pathology of the disease in that patients with PMF display a mobilisation of haemopoietic progenitors from the bone marrow and upto a 200-fold increase in the number of circulating CD34+ cells have been reported. [22, 42] It has been proposed that this increase results from several mechanisms including a reduced CXCR4 expression on CD34+ cells [43, 44] and disruption of the CXCR4/SDF-1 axis by a bone marrow proteolytic environment resulting from altered expression of proteases. [45, 46] Gene expression analysis of the consequences of Givinostat treatment, a drug with antiproliferative and proapoptotic activity against MPN cells, demonstrated that of the genes associated with haemopoiesis shown to change, 25% were associated with motility and adhesion. [47] The consequences of MPL W515L expression on this process at a protein level have been elucidated in this study.

Although the mechanism underlying myelofibrosis associated with JAK2 (a MPL W515L stimulated target) induced MPNs has been reported to be the excess production of TGFβ by CD34+ cells [48] the importance of TGFβ to the pathogenesis of myelofibrosis and its utility as a therapeutic target in treatment of JAK2 induced MPN are still not clear. For example rather than a direct effect on fibroblasts it has been suggested that TGFβ promotes tumorigenesis by the metabolic reprogramming of the tumour microenvironment changing the mitochondrial activity of adjacent cancer cells. [49] Here we show that MPL W515L expressing cells display a profound induction of TGFβ secretion and that this TGFβ release is involved in the increased chemokinesis observed. Further we show that this TGFβ induced chemokinesis is achieved *via* S1P and the phosphorylation of THOC5 a protein involved in RNA processing and export. Given the increase in circulating CD34+ cells in the peripheral blood of MPN patients and the importance of S1P in the egress of HSCs from the bone marrow [11, 36] these observations are of clinical relevance.

Several reports link S1P to leukemia [50], [51] and it has very recently been reported that sphingosine kinase plays an oncogenic role in acute lymphoblastic leukemia by regulating MYC expression. [52] Here we extend these observations by not only showing that S1P plays a role in MYC expression but does so *via* THOC5 phosphorylation and potentially contributes to leukemogenesis by altering the motile behavior of the cells. Given our findings that changes in protein expression are not directly linked to changes in mRNA expression [13, 14] and THOC5 binds MYC mRNA [20] it is noteworthy that Jiang et al [53] demonstrated that overexpression of SPK led to an increase in MYC expression *via* induction of MYC mRNA translation. It is therefore possible to hypothesize since THOC5 phosphorylation is a cytoplasmic event that it is linked to mRNA translation rather than mRNA transport. It is also interesting to speculate that the observed increase in S1P is related to the reported JAK2 activated changes in PP2a [54] which has been shown to selectively eradicate drug-resistant CML stem cells [55]. These and other data [19] are developing an understanding of THO complex modulation by leukemogenic oncogenes and also chemotactic factors that control stem cell retention in the marrow.

In conclusion our analysis of the effects of the leukemogenic oncogene MPL W515L has revealed effects on motility which are linked to MYC and TGFβ expression, S1P production and THOC5 phosphorylation hence offering novel potential targets for therapeutic intervention. We have illustrated the potential of a combination therapy of MYC and sphingosine kinase inhibition to preferentially kill HSCs from MPL W515L positive patients and we are in the process of assessing the utility of S1P, TGFβ and MYC inhibitors further. These studies will include a proteomic and transcriptomic assessment of drug action increasing our knowledge of MPLW515L driven malignancies. These studies will be undertaken alongside a more detailed analysis of THOC5 phosphorylation mediated control of MYC expression which will allow the identification of further treatment strategies. In addition we are utilising our cell line model to identify drugs capable of preventing the up-regulation of TGFβ production with a view to preventing fibrosis. All this knowledge will be used to inform any in vivo studies undertaken.

## MATERIALS AND METHODS

### Cell lines and mass spectrometry

Ba/F3 cells were transfected with MSCV retroviral vectors and maintained as previously described. [13] Cellular fractionation was undertaken with a kit from Active Motif (Belgium) with modifications as previously described. [15] Isobaric tagging using 8 channel ITRAQ^TM^ reagent and nanoflow liquid chromatography plus tandem mass spectrometry were performed as described previously. [15] Data was processed by a ‘Thorough’ search against the Ensembl (FIXME, www.ensembl.org) mouse database (release 58) using ProteinPilot 3 software (Paragon version 3.0.0.0, 113442, SCIEX) with default settings including the allowance of one missed/nonspecific cleavage, MMTS and 8 plex iTRAQ modifications. All the data was normalised such that the median was 1.0 and the ratios checked to ensure they had a normal distribution (Supplementary Figure 1). Sphingosine-1-phosphate was extracted and analysed by liquid chromatography coupled to electrospray ionisation tandem mass spectrometry (LC/ESI-MS/MS) as previously described. [56]

### Protein measurement

Western blot analysis was performed using standard protocols and images acquired using Quantity One software (BioRad, UK). Antibodies used are shown in Supplementary Table 1. Cell surface expression of specific proteins was assessed using flow cytometry with the LSRFortessa cell analyzer (Becton Dickinson, UK) and data analysed with FlowJo (Tree Star, USA) software as previously described. [19] Cells were stained with either APC rat-anti mouse CD184/CXCR4 or PE Rat anti-mouse CD45 (Becton Dickinson). TGFβ concentrations in cell culture supernatants were measure using the “Quantikine” ELISA kit (R&D Systems, UK) as per manufacturer's instructions.

### Cell motility assays

Chemotaxis assays were performed as previously described [12] using a Boyden chamber assay. Primary cell work was carried out in a 96 well plate format (Neuroprobe, USA) and cell line work in 24 well plate format (Sigma Aldrich, UK). The number of cells migrating in response to 200ng/ml CXCL12 was assessed over 6 hours. Where indicated cells were pre-incubated with inhibitors for 2 hours.

### siRNA transfection

Cells were transiently transfected with the 260nM MISSION^®^ esiRNA targeting mouse MYC (Sigma Aldrich) or a negative control siRNA (Silencer Select Negative Control 2, Ambion, UK). Transient transfection was achieved using the Amaxa Nucleofector II device (Kit V, Program X-001) as per manufacturer's instructions (Lonza, Switzerland). Following transfection the cells were incubated at 37°C in a 5% (v/v) CO_2_ in air incubator for 48 hours before being used in motility assays and protein expression assessed *via* western blot.

### Patient material

Use of human tissue had ethical approval from the NRES committee of the regional NHS health research authority (14/LO/0489). Primary MPL W515L positive samples from patients with Essential Thrombocythemia were obtained from the Cambridge Blood and Stem Cell Biobank. The CD34^+^ population was enriched using CliniMACS (Miltenyi Biotec) according to standard protocols. Control samples were peripheral blood samples obtained from patients with no known diseases. Colony forming assays were performed by plating CD34^+^ cells in Methylcellulose complete media (R&D systems) supplemented with 2u/ml EPO at a density of 3000cells/ml. Plates were incubated at 37°C in 5% CO_2_ /5% O_2_ for 7 days before the number of colonies were counted.

## SUPPLEMENTARY MATERIALS TABLES AND FIGURES






