# Supplementary material for: MPL W515L expression induces TGFβ secretion and leads to an increase in chemokinesis via phosphorylation of THOC5
Source: Oncotarget. 2016 Feb 23;7(10):10739–55. doi: 10.18632/oncotarget.7639 (PMC4905435; doi:10.18632/oncotarget.7639)
Supplement: Supplementary file 1 [file oncotarget-07-10739-s001.pdf]

MPL W515L expression induces TGFβ secretion and leads to an increase in chemokinesis *via* phosphorylation of THOC5

Supplementary Materials

Supplementary Table 1: Antibodies used in the study.

Antibody	Company	Dilution factor
α tubulin	Santa Cruz (sc-5286)	1/1000
anti phosphotyrosine (4G10)	Upstate (#05-321)	1/1000
β-actin	Sigma-Aldrich (A5060)	1/500
CD45	BD Pharmingen(610266)	1/500
CXCR4	Santa Cruz (sc-6190)	1/200
Fyb	Upstate (07-546)	1/1000
lamin A/C	Cell Signaling Technology(#2032)	1/1000
Lyn	Santa Cruz (sc-28790)	1/500
MYC	Cell Signaling Technology(#2276)	1/500
phospho S465/467 SMAD2	Cell Signaling Technology(#3108)	1/500
phospho Y225 THOC5	Eurogentec	1/750
SKAP2	Santa Cruz (sc-9178)	1/500
SMAD2	Cell Signaling Technology(#5339)	1/1000
SMAD7	Invitrogen (42-0400)	1/100
THOC5 (F6D/11)	LLR Immunodiagnostic Unit	1/2
TpoR	Abcam (ab172061)	1/100

Supplementary Table 2: 95% significance interval.

	95% significance interval defining a change			
	Run1	Run 2	Run 3	Technical repeat of Run 3
Nuclear proteome	>1.4 to < 0.7	>1.6 to < 0.6	>1.5 to < 0.7	>1.3 to < 0.8
Cytoplasmic Proteome	>1.3 to < 0.7	>1.4 to < 0.7	>1.6 to < 0.6	No run undertaken

Table showing the ratios employed to define a change. These are the values that 95% of ratios for the internal replicate lie between. This "significance interval" is individually determined for each experimental run and attempts to account for the technical and biological variation seen in each run.

Supplementary Table 3: All cytoplasmic proteins identified.

Table showing all 3550 nuclear proteins identified across the three replicate experiments of Ba/F3 cells transfected with MPLW515L. The first four columns display protein and gene accessions, gene symbol and name. The next nine columns give information from the search results for the identification of the protein in each experiment (blanks if not identified). ‘’Coverage’’ indicates what percentage of the protein sequence was identified. ‘’Unique peptides’’ indicates the number of unique peptides discovered whilst ‘’Peptides used’’ indicates the number of unique peptides used to derive the quantification. The remaining columns display quantification ratios (against control) and associated p-values and error factors for each replicate. Data consists of 43,818 peptides with 22,903 having associated quantification.

Supplementary Table 4: All nuclear proteins identified.

Table showing all 3392 nuclear proteins identified across three biological replicate experiments and one instrument technical replicate of Ba/F3 cells transfected with MPLW515L. The first four columns display protein and gene accessions, gene symbol and name. The next twelve columns give information from the search results for the identification of the protein in each experiment (blanks if not identified). ‘’Coverage’’ indicates what percentage of the protein sequence was identified. ‘’Unique peptides’’ indicates the number of unique peptides discovered whilst ‘’Peptides used’’ indicates the number of unique peptides used to derive the quantification. The remaining columns display quantification ratios (against control) and associated p-values and error factors for each replicate. The data includes 3 biological replicates and one instrumentation technical replicate. Data consists of 53,336 peptides with 27,895 having associated quantification.

A

B

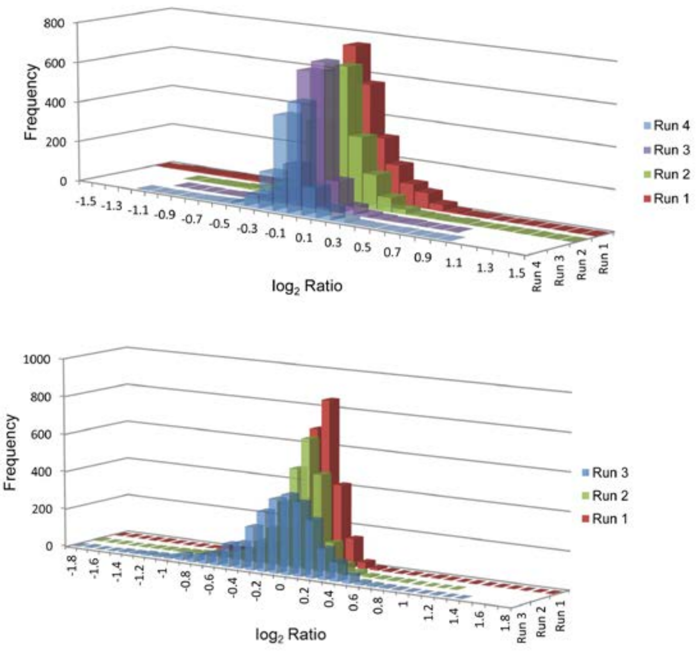

Supplementary Figure 1: Distribution of protein quantification ratios

Distribution of protein quantification ratios for all nuclear (Figure 1A) and cytoplasmic (Figure 1B) proteins identified and quantified. The histograms show the distribution of (log) ratios for the proteins in the three biological replicate (Figure 1B) and three biological replicate an instrument technical repeat (Figure 1A).

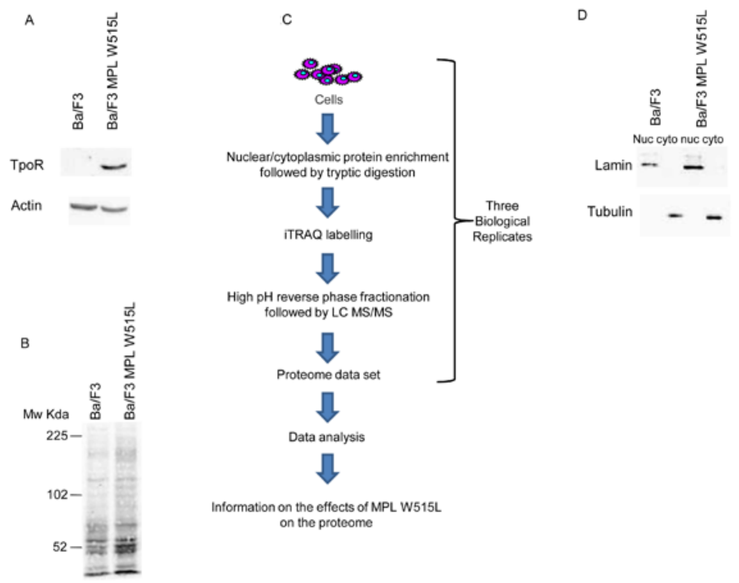

Supplementary Figure 2: Cell Line and subcellular fractionation QC.

Western Blot analysis of Ba/F3 whole cell lysates with anti- thrombopoietin receptor (Figure 2A) and anti-phosphotyrosine antibody (Figure 2B) in control and MPL W515L transfected Ba/F3 cells. Figure 2C; A schematic representation of the experimental workflow. Figure 2D; Assessment of the quality of the nuclear cytoplasmic fractionation using lamin (nucleus) and tubulin (cytosol) as markers following isolation nuclear and cytoplasmic lysates were separated by SDS PAGE and the distribution of these entities assessed by western blot analysis.

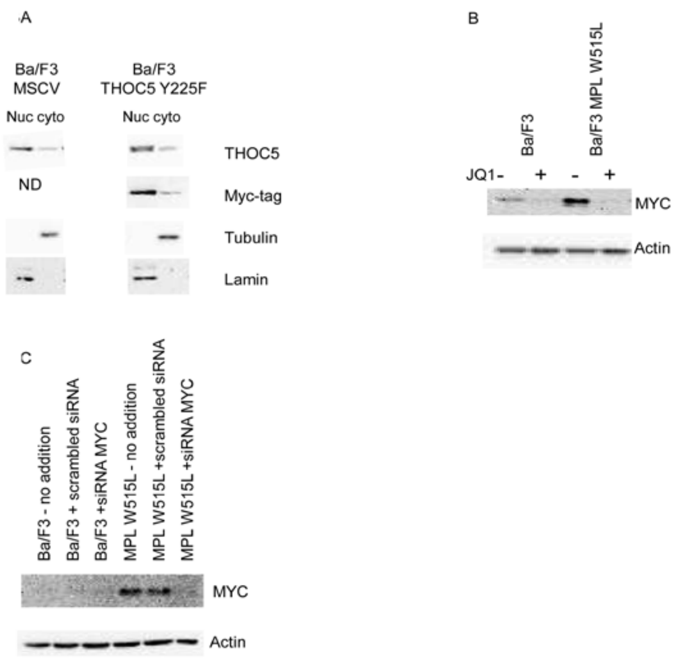

Supplementary Figure 3: THOC5 Y225F distribution and confirmation of MYC downregulation.

Figure 3A: Control and THOC5 Y225F (myc-tagged) expressing cells were subject to nuclear/cytoplasmic fractionation and the levels and distribution of the proteins indicated assessed by western blot analysis. Lamin and tubulin expression were used as loading controls and fractionation markers. Western blot analysis of MYC expression in Ba/F3 and MPL W515L expressing cells following 6 hour treatment with 500nM JQ1 (Figure 3B) or 48 hours post transfection with siRNA targeting MYC or a scrambled control (Figure 3C).

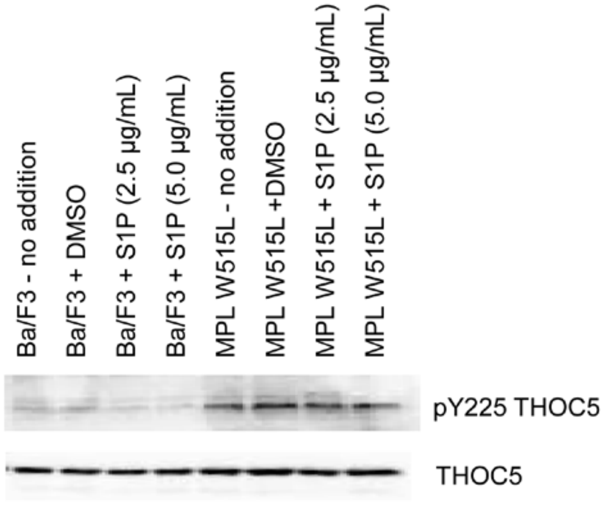

Supplementary Figure 4: S1P effects on THOC phosphorylation.

Western blot analysis of THOC5 and phospho Y225 THOC5 expression in Ba/F3 and MPL W515L expressing cells following 6 hour treatment with either DMSO, as a carrier control, or 2.5 and 5µg/ml S1P.
